# 5-HT3 and 5-HT4 antagonists inhibit peristaltic contractions in guinea-pig distal colon by mechanisms independent of endogenous 5-HT

**DOI:** 10.3389/fnins.2013.00136

**Published:** 2013-08-05

**Authors:** Tiong C. Sia, Malcolm Whiting, Melinda Kyloh, Sarah J. Nicholas, John Oliver, Simon J. Brookes, Phil G. Dinning, David A. Wattchow, Nick J. Spencer

**Affiliations:** ^1^Discipline of Human Physiology and Center for Neuroscience, Flinders UniversityAdelaide, SA, Australia; ^2^Department of Medical Biochemistry, Flinders UniversityAdelaide, SA, Australia; ^3^Department of Surgery, Flinders UniversityAdelaide, SA, Australia

**Keywords:** serotonin antagonists, peristalsis, colon, transit, motility, peristaltic reflex

## Abstract

Recent studies have shown that endogenous serotonin is not required for colonic peristalsis *in vitro*, nor gastrointestinal (GI) transit *in vivo*. However, antagonists of 5-Hydroxytryptamine (5-HT) receptors can inhibit peristalsis and GI-transit in mammals, including humans. This raises the question of how these antagonists inhibit GI-motility and transit, if depletion of endogenous 5-HT does not cause any significant inhibitory changes to either GI-motility or transit? We investigated the mechanism by which 5-HT3 and 5-HT4 antagonists inhibit distension-evoked peristaltic contractions in guinea-pig distal colon. In control animals, repetitive peristaltic contractions of the circular muscle were evoked in response to fixed fecal pellet distension. Distension-evoked peristaltic contractions were unaffected in animals with mucosa and submucosal plexus removed, that were also treated with reserpine (to deplete neuronal 5-HT). In control animals, peristaltic contractions were blocked temporarily by ondansetron (1–10 μM) and SDZ-205–557 (1–10 μM) in many animals. Interestingly, after this temporary blockade, and whilst in the continued presence of these antagonists, peristaltic contractions recovered, with characteristics no different from controls. Surprisingly, similar effects were seen in mucosa-free preparations, which had no detectable 5-HT, as detected by mass spectrometry. In summary, distension-evoked peristaltic reflex contractions of the circular muscle layer of the guinea-pig colon can be inhibited temporarily, or permanently, in the same preparation by selective 5-HT3 and 5-HT4 antagonists, depending on the concentration of the antagonists applied. These effects also occur in preparations that lack any detectable 5-HT. We suggest caution should be exercised when interpreting the effects of 5-HT3 and 5-HT4 antagonists; and the role of endogenous 5-HT, in the generation of distension-evoked colonic peristalsis.

## Introduction

Since the early 1950's, endogenous serotonin release from the gastrointestinal (GI) tract has been suggested to play an important role in the control of different GI motility patterns, such as peristalsis, in the small and large intestine (Büllbring and Lin, 1957, 1958; Büllbring et al., 1958; Grider et al., 1996; Jin et al., 1999; Heredia et al., 2009). Evidence to support this hypothesis comes from the fact that the largest quantity of serotonin in the body is synthesized in enterochromaffin cells (Erspamer, 1954) and high concentrations of 5-HT can be dynamically released from the mucosa (Bertrand, 2006; Keating and Spencer, 2010; Spencer et al., 2011). In addition, it is well known that exogenous 5-HT potently stimulates GI-motility (Büllbring and Lin, 1957, 1958; Keating and Spencer, 2010; Spencer et al., 2011) and a variety of antagonists of 5-HT receptors can potently inhibit, or block peristalsis, reduce transit (Grider et al., 1996; Kadowaki et al., 1996; Heredia et al., 2009) and rectal distension reflexes (Shimatani et al., 2003).

Despite significant circumstantial data supporting a role for endogenous 5-HT in the control of GI-motility, the notion that endogenous 5-HT plays a major role in control of GI-motility was substantially revised in the past couple of years, based on findings from independent laboratories (Yadav et al., 2010; Li et al., 2011; Spencer et al., 2011; Sia et al., 2013). These recent studies showed that when 5-HT was depleted from enteric neurons; followed by removal of the mucosa and submucosal plexus, distension-evoked peristalsis still occurred without any significant deficits (Sia et al., 2013). Similar findings have now been reported for colonic migrating motor complexes (CMMCs) in the mouse colon (Spencer et al., 2013). Coincidently, recent findings from the laboratory of Gershon and colleagues (Yadav et al., 2010; Li et al., 2011), demonstrated that deletion of the gene responsible for 5-HT synthesis in enterochromaffin (EC) cells led to no inhibitory effects on GI-transit in live mice (Yadav et al., 2010; Li et al., 2011). These recent findings are important because the vast majority of 5-HT in the body (>95%) is synthesized within the intestinal mucosa, with only minor quantities synthesized in the enteric nervous system. In fact, less than 1% of enteric neurons have been shown to synthesize 5-HT (Costa et al., 1996). Also, 5-HT-mediated synaptic potentials are rarely recorded from myenteric neurons (Galligan et al., 2000; Nurgali et al., 2003; Furness, 2006). When they have been reported, they occur in a very small proportion of enteric neurons and are of small amplitude (Galligan et al., 2000; Nurgali et al., 2003; Furness, 2006). Studies have shown that 5-HT-mediated fast synaptic potentials are extremely rare in guinea-pigs (Bornstein et al., 2004; Furness, 2006); and have never been detected in the mouse colon (Furukawa et al., 1986; Nurgali et al., 2004), rat small intestine (Brookes et al., 1988) or human colon (Brookes et al., 1987). In these species, all fast synaptic potentials in enteric neurons are abolished by nicotinic antagonists, with no serotonergic fast synaptic potentials being reported. Of particular interest is why do 5-HT receptor antagonists potently inhibit GI motility and reduce transit in these same species where no serotonergic fast EPSPs have been detected (Balfour et al., 2000; Bush et al., 2001)?

Whilst there is no doubt that antagonists of 5-HT3 and 5-HT4 receptors can delay GI transit in both humans and laboratory animals, the mechanisms by which these drugs induce their inhibitory effects on GI-motility is poorly understood. Recently, we reported that 5-HT3 and 5-HT4 receptors antagonists had no significant effect on colonic peristalsis in guinea-pig distal colon, when peristalsis was evoked by acute luminal distension using fecal pellets, or slow intraluminal fluid distension (Sia et al., 2013). These results were inconsistent with data obtained from other laboratories where it was shown that the same 5-HT3 and 5-HT4 antagonists (at the same concentrations) potently inhibited peristalsis, when evoked by the same stimuli in the same preparation (Kadowaki et al., 1996; Jin et al., 1999). In the current study, we used a different stimulus to evoke a cyclical peristaltic contraction. We used maintained distension of the colonic wall, using an artificial fecal pellet that was not free to be propelled along the colon. This is an entirely different type of distension stimulus to that applied in our recent study (Sia et al., 2013). The primary aim of this study was to determine whether antagonists of 5-HT3 and 5-HT4 receptors can inhibit distension-evoked peristaltic contractions, evoked by maintained localized intraluminal distension, and if so, what effects would antagonists of 5-HT3 and 5-HT4 receptors have in preparations that were depleted of all endogenous 5-HT?

## Methods

### Preparation of tissues

Adult male guinea pigs, weighing between 350–450 g, were killed by a blow to the occipital region and exsanguinated, in a manner approved by the Animal Welfare Committee of Flinders University. The distal colon (5–10 cm from the anus) was removed and placed in warm Krebs solution which was constantly bubbled with carbogen gas (95% O_2_/5% CO_2_). After a period of time (usually <20 min), fecal pellets naturally present were expelled from the colon. A segment of distal colon (6 cm in length) was mounted in an organ bath and left to equilibrate for 30 min. After this time, mechanical recordings were made from the circular muscle using the protocol described below.

### Technique to deplete endogenous 5-HT from the enteric nervous system

The enteric nervous system was depleted of endogenous 5-HT using the technique first demonstrated by Costa and Furness (Costa et al., 1982; Sia et al., 2013). This involves a single subcutaneous injection of reserpine (at a concentration of 0.5 mg/Kg) between 18 and 24 h prior to euthanasia. 5-HT immunoreactivity is not detected in the enteric nervous system after this procedure (Costa et al., 1982). However, because reserpine does not deplete 5-HT from the mucosa, we further employed our recently published method (Spencer et al., 2011) to remove the mucosa, submucosa and submucosal plexus by inverting the colon and using sharp dissection. This allows us to test whether acute depletion of 5-HT from enteric nerves and the absence of the mucosa and submucosal plexus impairs colonic motility, without potential complications such as compensation induced in genetically modified animals.

### Terminology used to define different preparations

Throughout the results we refer to “control” preparations as those which were not treated with reserpine and had their mucosa and submucosal plexus intact. We refer to “reserpine treated—mucosa present” preparations as those which have been treated with reserpine and which also have their mucosa and submucosal plexus present. Preparations that had been treated with reserpine but also had their mucosa and submucosal plexus removed are referred to as “reserpine-treated, mucosa removed” preparations.

### Mechanical recordings of circular muscle contractility during peristalsis evoked by a fixed fecal pellet

We recorded the isometric force generated by the circular muscle during each peristaltic contraction by inserting an artificial fecal pellet into the oral end of colon and allowing the pellet to naturally propagate midway along the length of colon (Spencer et al., 2011). The pellet was attached to fine surgical cotton thread that was then fixed in the mid region of the preparation so that it could not be expelled. Cyclical peristaltic contractions were recorded with an isometric recording transducer Grass (FT-03C; Grass, Quincy, MA, USA) connected to the ligature. These experiments were described in the results section as the maintained distension experiments induced by a fixed pellet, since the pellet was not free to move along the colon. The isometric force transducers were connected to two custom made preamplifiers (Biomedical engineering, Flinders University) and then to a Powerlab (model: 4/30; AD Instruments, Bella Vista, NSW, Australia). Labchart version 6.0 (AD Instruments, Australia) was used for analysis of data.

### Measurements and statistics

Measurements of the peak amplitude of each peristaltic contraction were measured from tension recordings, as was the interval between each cyclical peristaltic contraction. Data in the results section are presented as means ± S.E.M. The use of “N” in the results section refers to the number of animals on which observations were made. Data sets were considered statistically significant if *P* < 0.05. Students unpaired *t*-test was used to compare data.

### Immunohistochemistry

Isolated segments of guinea-pig distal colon were fixed by pinning sheet preparations of colon under constant tension in a Sylgard lined Petri dish (Dow Corning Corp., Midland, MI, USA) and immersing overnight in Zamboni's fixative (5% Formaldehyde and 15% saturated picric acid in 0.1 M phosphate buffer; pH 7.2) at 4°C. Preparations were then cleared in dimethyl sulfoxide (10 min immersion, repeated three times), tissue was washed in phosphate buffered saline (PBS); (0.2 M sodium phosphate buffer, pH 7.2) and a whole mount of the myenteric plexus and longitudinal muscle was prepared by removing the mucosa, submucosa and circular muscle with the aid of a dissecting microscope. Goat 5-HT antisera (Immunostar, Cat: 20079) was applied at 1:1500 overnight at room temperature then washed 3 × 10 min in PBS. Tissue was then incubated for a further 2 h in secondary antisera (Donkey anti Goat CY3; Jackson Immunoresearch Laboratories Inc) at 1:400 then washed 3 × 10 min in PBS and mounted in bicarbonate- buffered glycerol (pH 8.6).

### Drugs and solutions

The Krebs solution used contained (in mM): NaCl, 118; KCl, 4.7; NaHPO_4·_2H_2_0, 1.0; NaHCO_3_, 25; MgCl_·_6H_2_0, 1.2; D- Glucose, 11; CaCl_2·_2H_2_0, 2.5. Ondansetron hydrochloride and SDZ-205–557 were obtained from Sigma Chemical Co. St. Louis. Mo. USA, and were made up at a stock concentration of 10^−2^ M.

### Chemical analysis of tissue extracts for 5-HT by tandem mass spectrometry

The amount of 5-HT present in tissues was measured using solid-phase extraction, reversed-phase liquid chromatography and electrospray tandem mass spectrometry (LC-MSMS) with isotope dilution, essentially as described by Tareke et al. (2007). Liquid chromatography was performed using an Atlantis T3 column (3 μm particle size, 150 × 2.1 mm, Waters), while mass spectrometry used a 3200 Q-Trap instrument (ABSCIEX Toronto, Canada) tuned to measure ion transitions with m/z 177 > 160 for 5-HT, and 181 > 164 for tetra-deuterated 5-HT as internal standard. Quantitation was carried out using peak area ratios and Analyst v. 1.5 software with linear calibration plots covering the sample concentration range 0–1000 nmol/L. The limit of quantitation (S/N 5) was 0.5–1 nmol/L.

## Results

**Figure 1:**
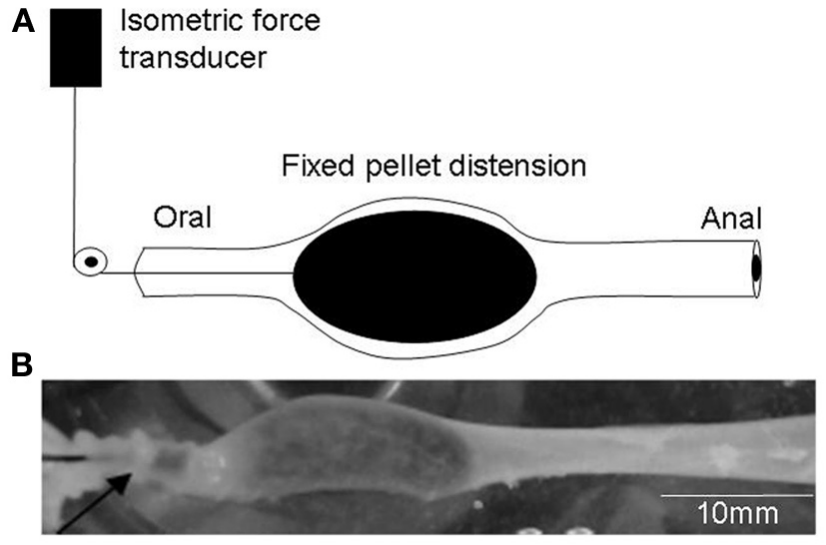
**Schematic representation of the method used to evoke repetitive peristaltic contractions**. **(A)** An artificial fecal pellet is inserted into the oral end of the isolated colon attached to fine cotton thread. The pellet was allowed to naturally propel along the colon, until it reached a point midway along the colon where it was fixed and isometric force measurements made of the degree of tension generated on the pellet, during each cyclical peristaltic contraction. **(B)** Shows a photomicrograph of the pellet fixed in the colonic lumen. The arrow indicates a robust circular muscle contraction active on the oral side of the pellet.

We investigated the effects of maintained colonic wall distension by inserting an artificial fecal pellet into the oral end of colon. The pellet was free to propel anally to a point midway along the colon, at which point the pellet was fixed in place via fine suture thread, so that it could not be further expelled, as previously described (Spencer et al., 2011), (see Figure 1). In response to maintained distension of control animals with mucosa present, cyclical peristaltic contractions were evoked at a mean interval of 278.10 ± 62.3 s (*N* = 6), or reserpine-treated animals with mucosa and submucosal plexus removed (398.8 ± 124 s; *N* = 4; *P* = 0.15). This revealed that control mechanisms that regulated the frequency of peristaltic contractions were independent of the mucosa or submucosal plexus, or, endogenous 5-HT in enteric neurons. Similarly, the mean peak amplitude of circular muscle contractions induced during each peristaltic contraction was 10.64 ± 0.8 g in control animals, which, was no different from reserpine-treated animals with mucosa present (11.4 ± 0.8 g; *P* = 0.61; *N* = 16), or reserpine-treated animals with mucosa and submucosal plexus removed (9.7 ± 1.5 g; *P* = 0.07; *N* = 4).

### Effects of 5-HT3 or 5-HT4 receptor antagonist on distension-evoked peristaltic contractions

**Figure 2:**
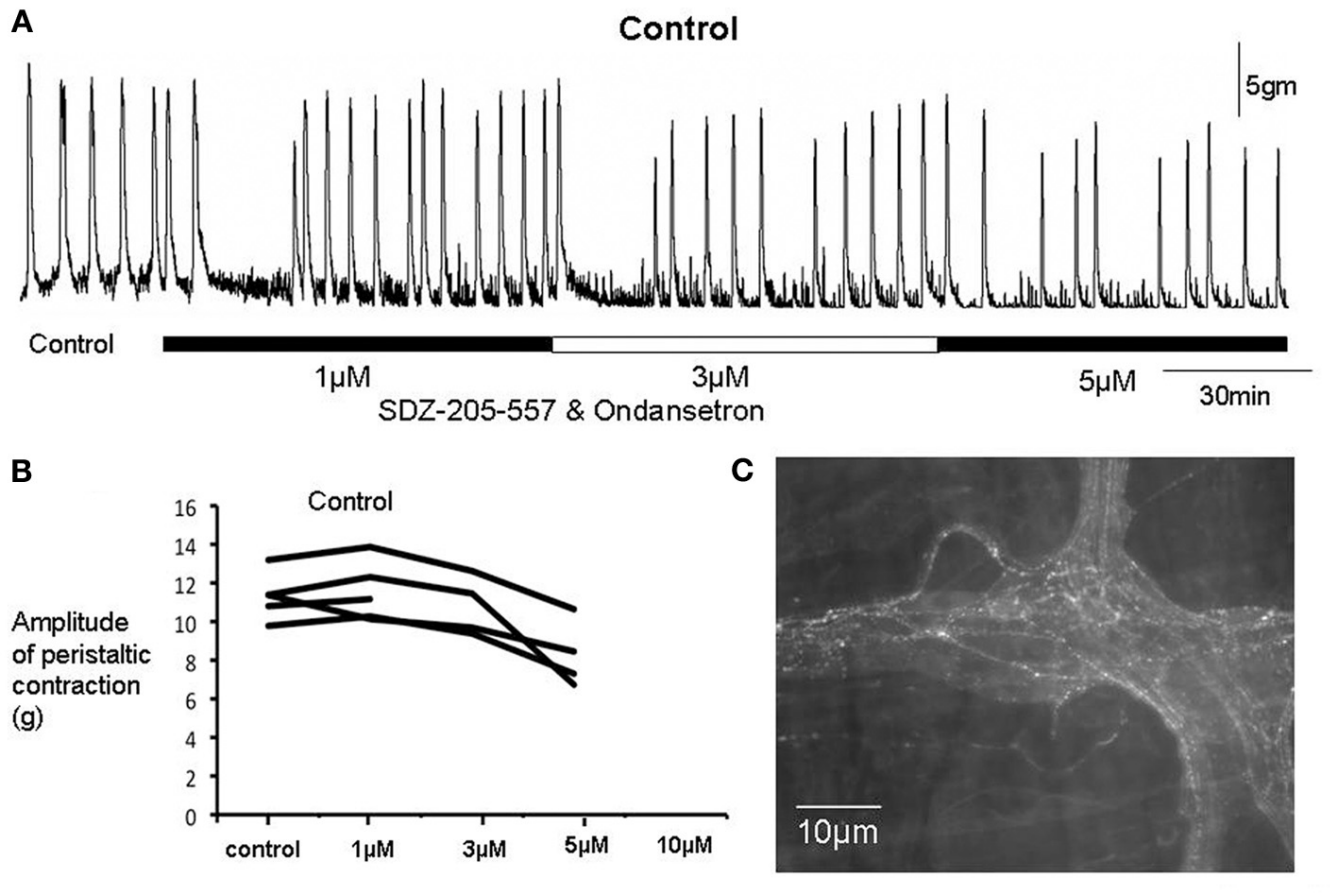
**Effects of ondansetron and SDZ 205-557 on a control colonic preparation**. **(A)** On each occasion when increasing concentrations of both antagonists were applied a temporary blockade of peristaltic contractions occurred. However, in the continued presence of both drugs, contractions recovered with characteristics not detectably different from controls. **(B)** Shows that when peristaltic contractions recovered at 1 and 3 μM, the amplitude of these contractions was not different from control prior to drug addition. **(C)** Immunohistochemical staining for 5-HT revealed the presence of immunoreactive varicose axons in internodal strands and ganglia.

**Figure 3:**
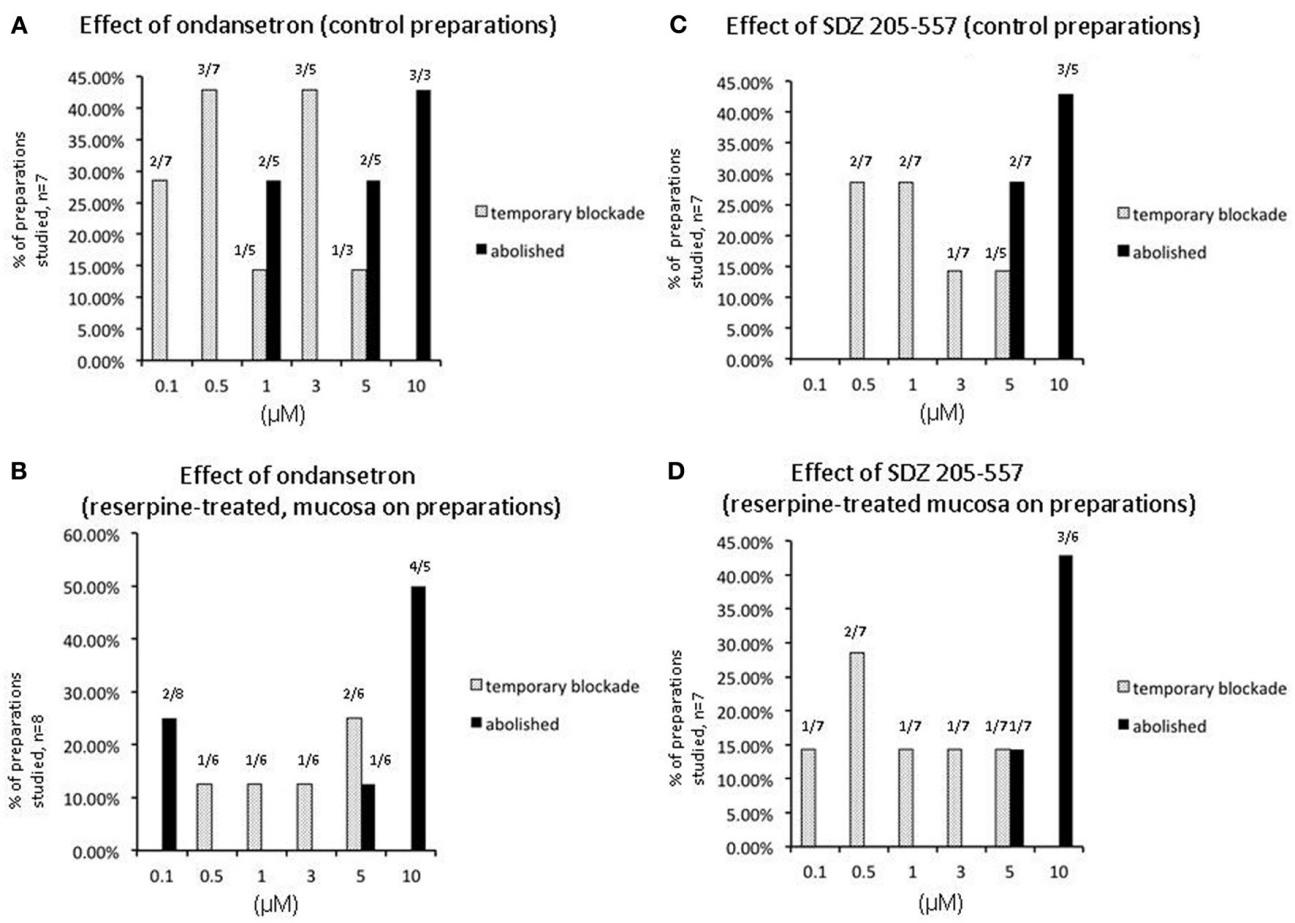
**Graph shows the number of preparations of colon from separate animals that showed a temporary or permanent blockade of peristaltic contractions in control preparations and preparations obtained from animals treated with reserpine prior to being euthanized**. **(A)** Shows control animals, a large proportion of preparations showed a temporary blockade of peristaltic contracions upon application of ondansetron at increasing concentrations of antagonists applied. **(B)** Similar results were obtained in reserpine-treated preparations with mucosa present. **(C)** Shows at increasing concentrations of SDZ-205–557 control preparations and reserpine-treated preparations showed a temporary blockade of peristaltic contractions **(D)**. At higher concentrations peristaltic contractions were blocked permanently.

**Figure 4:**
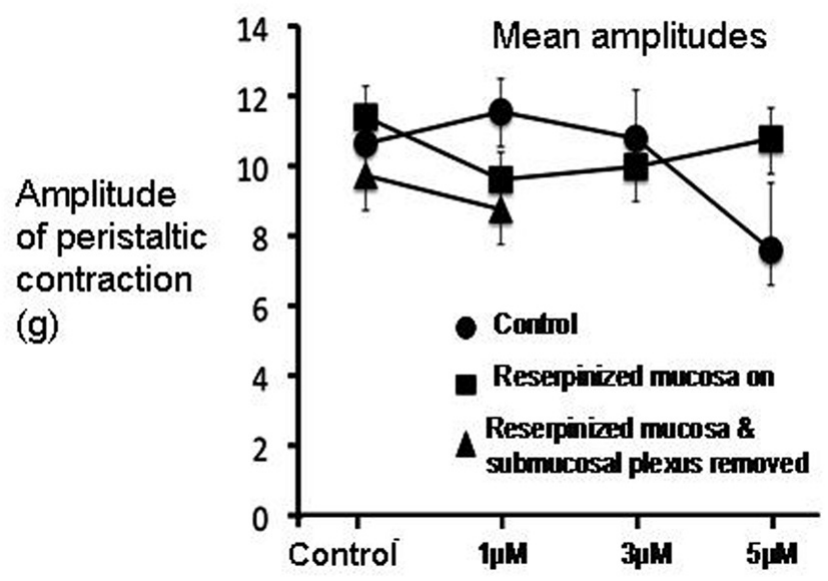
**Graph shows mean changes in amplitude of peristaltic contractions, in response to the combined application of ondansetron and SDZ-205-557 in three different experimental groups**. In control animals (see filled circles), after an initial blockade of peristaltic contractions by both antagonists, the amplitude of peristaltic contractions recovered to amplitudes that were not significantly different from reserpine-treated animals which had mucosa present (filled squares), or reserpine-treated that were mucosa-free (filled triangles).

**Figure 5:**
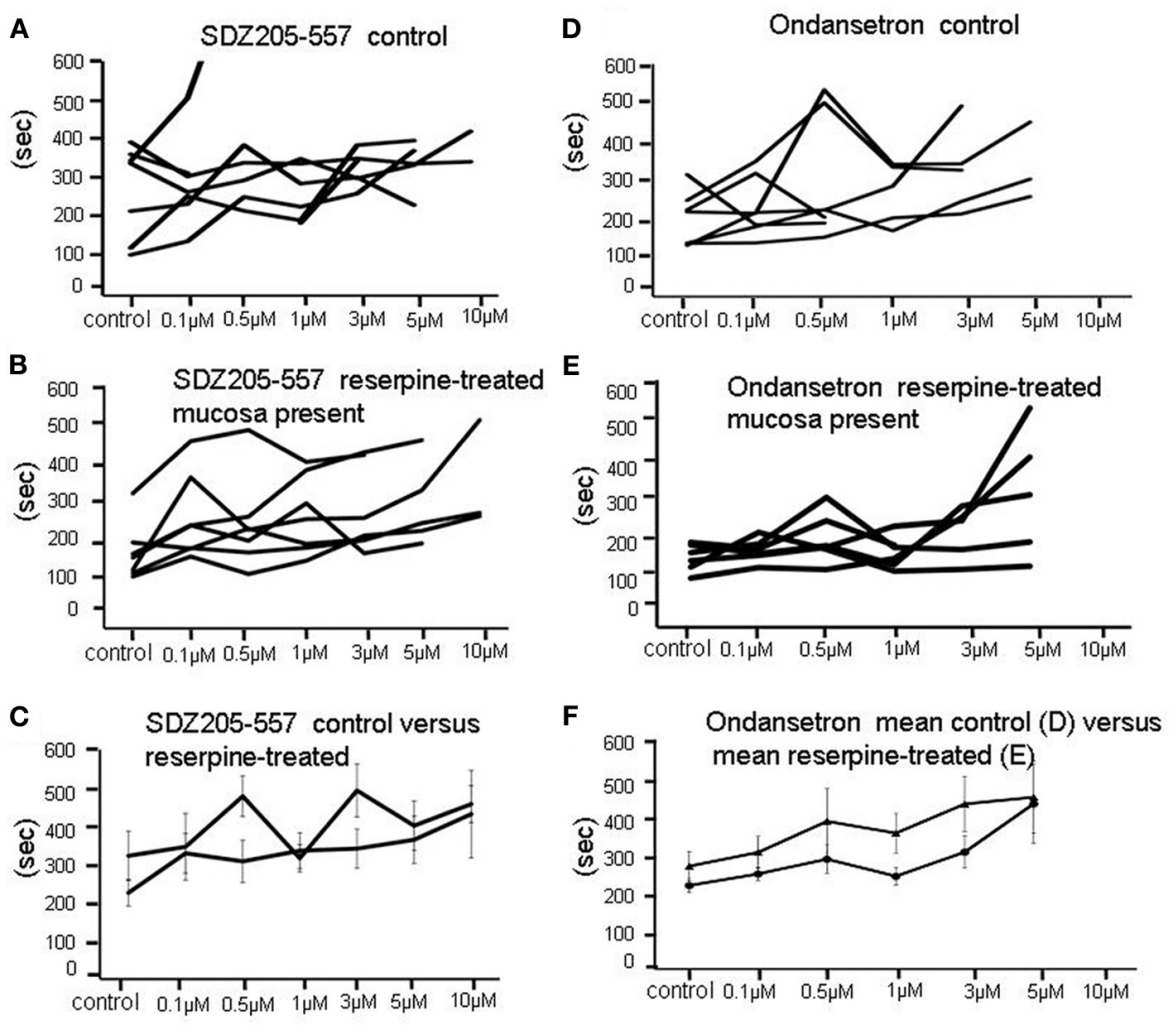
**Effects of application of SDZ-205-557 or ondansetron on the intervals between repetitive peristaltic contractions**. **(A)** Show a graphical representation of the responses of individual preparations of colon to SDZ-205-557. No clear changes are seen. **(B)** similar lack of effect of SDZ-205–557 on reserpine treated animals, in which the mucosa was present. **(C)** Shows the mean changes in interval between peristaltic contractions in control **(A)** and reserpine-treated **(B)** preparations overlaid on the same graph. **(D)** Shows the same experiment as in **(A)**, but ondansetron is used instead of SDZ-205–557. Again, no major changes in intervals were detected. **(E)** Shows the lack of effect of ondansetron on intervals between peristaltic contractions in reserpine-treated animals, with mucosa present. **(F)** Shows mean data of (**D** and **E**) superimposed. No overall significant effects were detected.

We were particularly interested in the effects of the selective 5-HT3 or 5-HT4 antagonists, ondansetron and SDZ 205–557, on repetitive peristaltic contractions evoked in control preparations; and in preparations that were treated with reserpine but retained mucosa. Initially, we applied ondansetron or SDZ 205–557 to control segments of colon, when reliable peristaltic contractions were evoked by maintained fixed pellet distension (e.g., Figure 1A). In most preparations, there was no effect of either antagonist (Figures 2, 3). However, in a small proportion of preparations it was found that application of either antagonist caused an initial blockade of peristaltic contractions (Figure 2). Surprisingly, in the continued presence of either of these antagonists, repetitive peristaltic waves recovered (Figures 2, 3), such that there was no significant difference in peak amplitude compared to controls (*P* > 0.05; unpaired Students *t*-test; *N* = 5; Figure 4). When these contractions recovered in the continued presence of either antagonist, the intervals between contractions were not significantly different from controls, suggesting that either receptor were not required for their generation (Figure 5). Only at excessively high concentrations between 5 and 10 μM was peristaltic contractions permanently abolished (Figure 3). Since most preparations were not affected by application of either antagonists applied alone, we were interested in applying both 5-HT3 and 5-HT4 antagonists at the same time, since it has been suggested that the combined application of a 5-HT3 and 5-HT4 receptor antagonist is required to block peristalsis (Grider et al., 1996; Kadowaki et al., 1996). Therefore, we tested the effects of both antagonists applied together.

**Figure 6:**
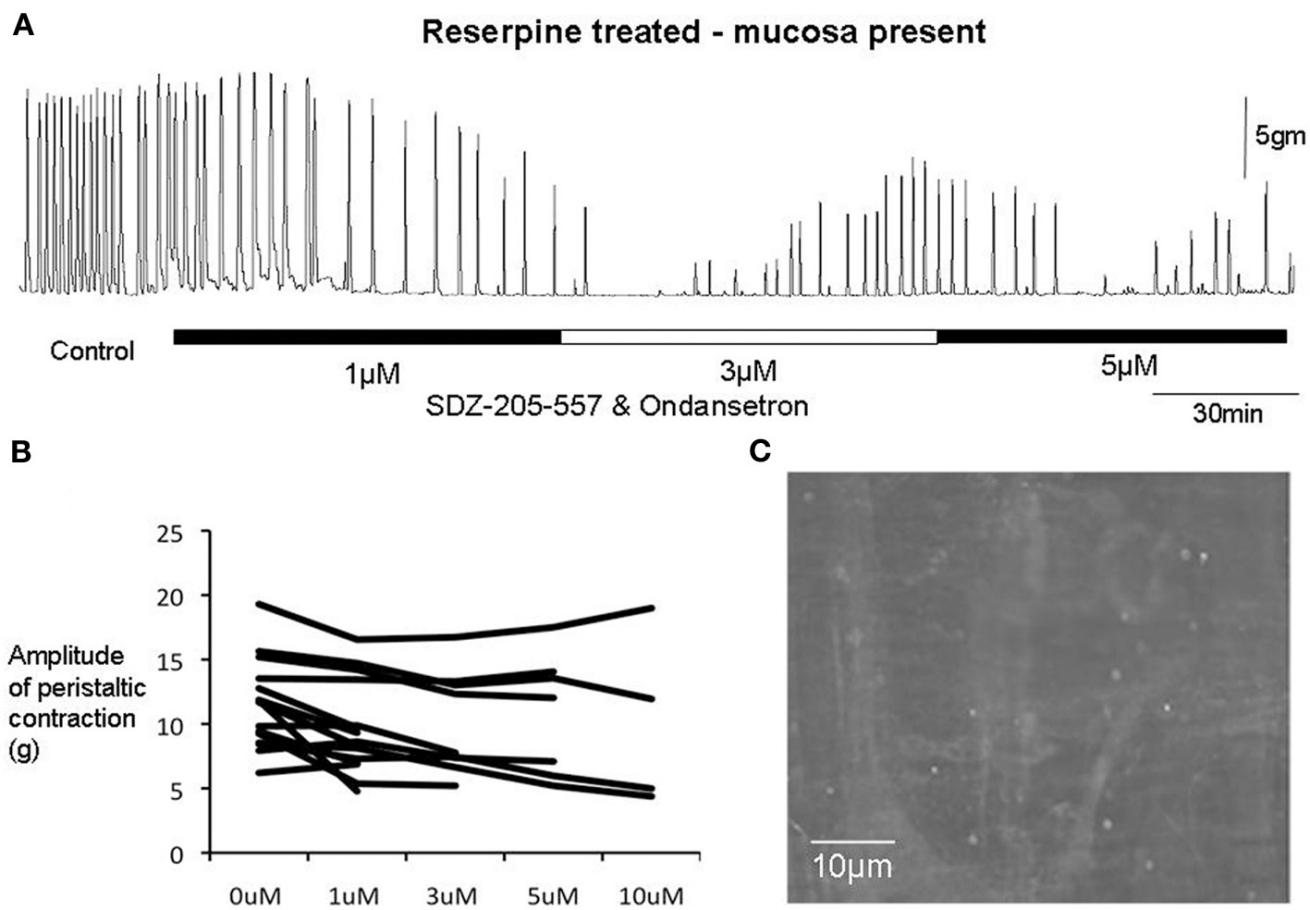
**(A)** In a reserpine-treated animal with mucosa present, combined application of SDZ-205-557 and ondansetron caused temporary inhibition of peristalsitic contractions which recovered even with increasing concentrations up to 5 μM. **(B)** Shows changes in amplitude of peristaltic contractions from individual animals. It is apparent that when these contractions recovers the amplitudes are unaffected in the majority of preparations, even up to 10 μM in concentration. **(C)** Shows that 5-HT has been depleted from myenteric ganglia in response to reserpine treatment.

When both ondansetron or SDZ 205–557 were applied together at either 1, 3, or 5 μM, similar results were obtained in preparations of colon from control animals; and reserpine-treated animals with intact mucosa and submucosal plexus (Figure 6). The primary effect of both these antagonists was a sudden blockade of repetitive contractions in a proportion of preparations studied (see Figure 3). The mean duration of the temporary blockade of both antagonists was 351.2 ± 109.3 s at 1 μM, 375.5 ± 52.1 s at 3 μM and 399.0 ± 70.3 s at 5 μM (Figure 6A). With regards to the amplitudes of peristaltic contractions following recovery from a temporary blockade, their peak amplitudes were also not different from control (*P* > 0.05; paired Students *t*-test; Figures 4, 6B). When peristaltic contractions recovered in the continued presence of these antagonists, the intervals between peristaltic contractions revealed similar findings. For example, in control animals, the average interval between peristaltic contractions prior to the application of antagonists was 278.0 ± 62.3 s (*N* = 5) and when they recovered in the presence of these antagonists, the mean interval was 232.4 ± 54.7 s, which was no different from control intervals (*P* = 0.86; *N* = 5). Similarly, following a temporary blockade at 3 μM, ondansetron and SDZ 205–557, the mean interval between peristaltic contractions which recovered was 219.1 ± 17.2 s (*N* = 5), but again this was not significantly different from the control interval between peristaltic contractions, prior to antagonist application (278.0 ± 62.3 s; *N* = 5; *P* = 0.68). Even increasing the concentration of both antagonists to 5 μM, the interval between peristaltic contractions during recovery (187.5 ± 50.8 s; *N* = 5) was not different compared to control conditions (278.0 ± 62.3 s; *N* = 4) prior to drug application (*P* = 0.87, *N* = 4). These observations strongly suggest that 5-HT3 or 5-HT4 receptor activation is not a prerequisite for distension-evoked peristaltic contractions to occur.

We incrementally raised the concentration of either ondansetron or SDZ-205–557 to determine the concentration of antagonists required to block peristaltic contractions. It was found to be similar between reserpine-treated (mucosa intact) animals and controls (Figure 3). Only at high concentrations of either antagonist between 5 and 10 μM was it possible to cause permanent blockade of activity in approximately half of animals studied (see Figure 3). It is noteworthy that ondansetron at 10 μM abolished more preparations (4 of 4 in control and 4 of 5 reserpine-treated, c.f. Figures 3A,B) compared with SDZ 205–557 alone (3 of 5 in control and 3 of 6 in reserpine-treated, c.f. Figures 3C,D). This means that even at 10 μM SDZ 205-557 approximately half of animals were not blocked by this excessively high concentration. We confirmed in reserpine-treated animals that myenteric ganglia were depleted of neuronal 5-HT, using immunohistochemical staining for 5-HT (Figure 6C).

### Effects of 5-HT3 and 5-HT4 receptor antagonist on peristaltic contractions evoked in preparations depleted of all endogenous 5-HT

**Figure 7:**
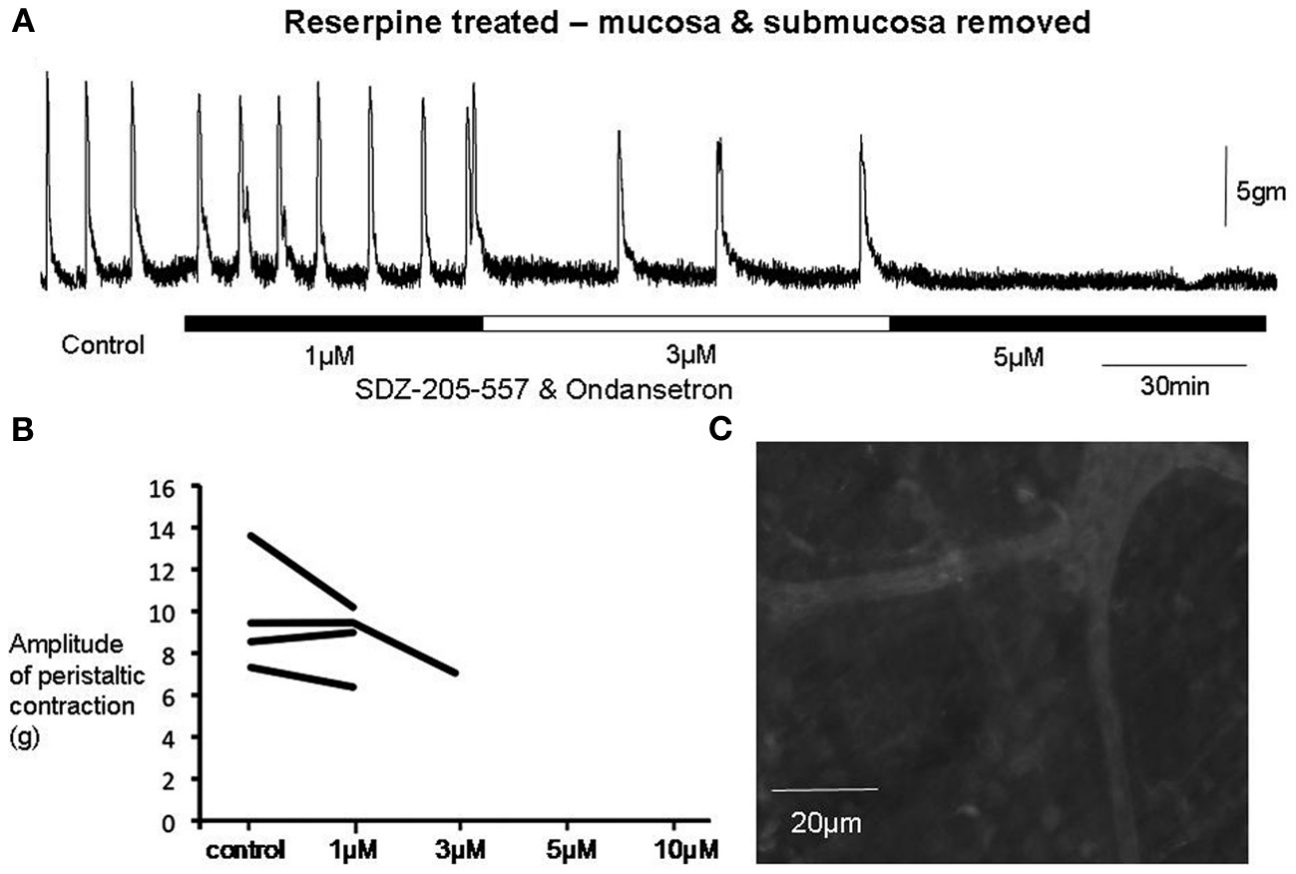
**(A)** In a reserpine-treated animal, with mucosa and submucosal plexus removed, the combined application of SDZ-205-557 and ondansetron had potent inhibitory effects on peristaltic contractions, even despite the lack of any 5-HT detected in these preparations with mass spectrometry or immunohistochemistry. The recording shows that peristalsis slows at 1 μM and is abolished at 3 μM. **(B)** Graph shows that 5-HT depleted preparations showed greater sensitivities to both antagonists. All preparations were permanently abolished by both antagonists at 3 μM. **(C)**, immunohistochemistry confirmed the absence of 5-HT in myenteric ganglia and internodal strands.

**Figure 8:**
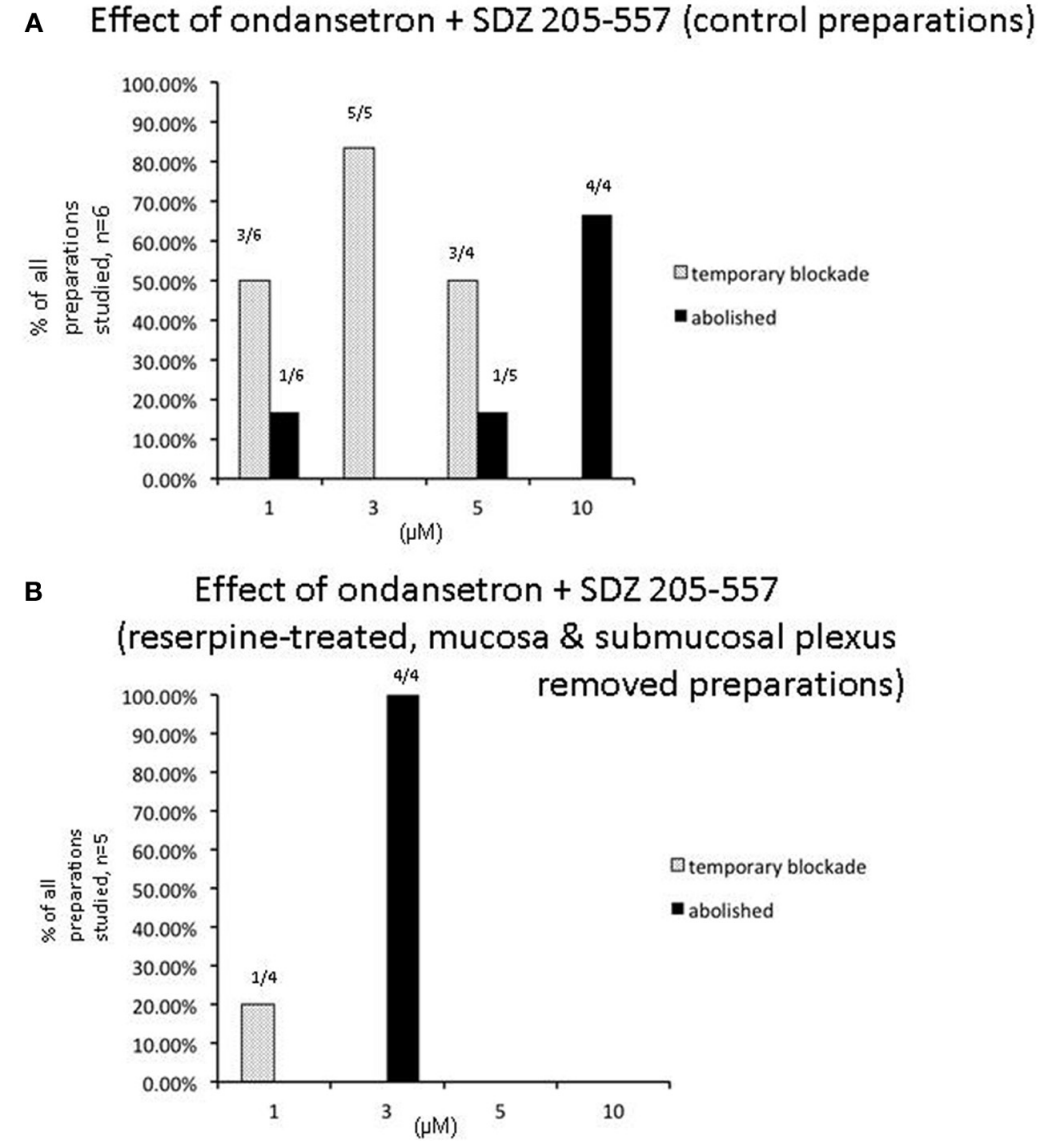
**Sensitivity of individual experiments to the combined effects of receptor antagonists to 5-HT3 and 5-HT4 receptors**. **(A)** Shows control data of the proportion of individual colonic preparations that showed repeated temporary blockade of repetitive peristaltic contractions with increasing concentrations of SDZ-205–557 and ondansetron. The table shows responses of 6 control animals to consecutive application of increasing concentrations of ondansetron and ondansetron (1–10 μM) and responses to 4 preparations treated with reserpine and also with mucosa and submucosal plexus removed. Interestingly, in reserpinized preparations, with mucosa and submucosal plexus removed, peristaltic contractions rarely recovered in the presence of both antagonists and most 3 of 4 animals were blocked at 1 μM, whereas in control animals, 4 out of 6 animals required 10 μM to permanently block these contractions. In **(A)**, it can be seen that 1 out 6 preparations was abolished at 1 μM of these antagonists, then increasing the concentration to 5 μM one additional preparation was blocked, then the remaining 4 preparations were all blocked at 10 μM. **(B)** In contrast, in reserpine-treated mucosa-free preparations, no preparations were blocked at 1 μM, but all were abolished at 3 μM.

Of particular interest to us was the combined effect of ondansetron and SDZ on preparations of colon that had been reserpine-treated and had their mucosa and submucosal plexus removed (Figure 7). These preparations had no detectable 5-HT using mass spectrometry, yet interestingly, application of both antagonists together still had a potent inhibitory effect on peristaltic contractions (Figure 7). One striking observation was that in reserpine-treated animals that also had their mucosa and submucosal plexus removed, combined application of ondansetron and SDZ 205–557 (1 μM) was actually more effective in abolishing these contractions (Figure 8A) than in control preparations. Fewer preparations showed a temporary blockade of contractions, followed by recovery. All contractions were abolished at 3 μM (Figure 8B). These major inhibitory effects on peristaltic contractions occurred, despite the fact that there was no detectable 5-HT in these preparations.

### Confirmation of depletion of 5-HT from enteric nerves using immunohistochemistry and mass spectrometry

Tandem-mass spectrometry was used to quantify the level of endogenous 5-HT in control colonic specimens and colonic specimens treated with reserpine. In control specimens, mass spectrometry was consistently able to detect 5-HT in concentrations between 1 and 10 nM (mean 1.9 ± 0.31 nM; *N* = 4). However, in reserpine-treated segments of colon, that also had their mucosa and submucosal plexus removed, mass spectrometry never detected the presence of serotonin to a sensitivity level of 0.5–1 nM (*N* = 4). This data was further independently verified using immunohistochemical labeling for 5-HT (Costa et al., 1982), in which control samples of colon always revealed the presence of 5-HT containing varicose fibers. However, in reserpine-treated animals, immunhistochemical labeling consistently revealed the absence of any endogenous 5-HT in internodal strands of myenteric ganglia (Figure 5D).

## Discussion

In this study, we evoked repetitive peristaltic contractions in the distal colon, by maintained intraluminal distension, using artificial fecal pellets that were fixed within the lumen. This is an entirely different method of colonic stimulation, compared to the approach we used in a recent study from our laboratory (Sia et al., 2013), where peristaltic contractions were evoked by insertion of fecal pellets that were free to move along the colon, or, by slow intraluminal fluid distension that could be expelled during each peristaltic wave. The major finding of the current study, is that 5-HT3 and 5-HT4 antagonists can have significant inhibitory effects on repetitive peristaltic contractions, evoked by maintained fecal pellet distension. However, these effects were usually temporary and in most preparations, peristaltic contractions recovered with normal motor characteristics, even despite continued exposure to the antagonists. Only at non-specifically high concentrations did these antagonists abolish peristaltic contractions and this effect occurred equally in 5-HT depleted preparations. The new information from this study is that exposure of increasing concentrations of 5-HT3 and 5-HT4 antagonists to the colon can cause repeated temporary blockade of peristaltic contractions of the circular muscle layer. These findings reveal substantial plasticity in the intrinsic neural circuitry underlying distension-evoked peristaltic reflex motor activity and leads us to propose that caution should be exercised when assuming a permanent blockade of motor activity has occurred upon initial exposure to 5-HT antagonist(s).

### Early hypotheses regarding the role of endogenous 5-HT in colonic peristalsis

It has been known for some time that a variety of 5-HT antagonists can have robust inhibit GI-transit and different motility patterns in the GI-tract. Despite these well-known inhibitory effects, there has been considerable speculation regarding the site of action and mechanisms by which these antagonists induce their effects. Equally unclear is the mechanism by which they inhibit GI-motility patterns, such as peristalsis or cyclical migrating complexes in the small or large intestine. One popular hypothesis has been that 5-HT antagonists could act to inhibit GI-motility by inhibiting the release of endogenous 5-HT from enterochromafiin (EC) cells in the mucosa, since these cell synthesize large quantities of 5-HT, or, by blocking serotonergic synaptic transmission in the enteric nervous system. The findings of the current study suggest this is unlikely to be the case, at least in the distal colon, since 5-HT3 and 5-HT4 antagonists were still found to inhibit peristaltic contractions in preparations of colon with no detectable 5-HT. Our data also shows that the primary mechanisms by which 5-HT3 and 5-HT4 antagonists inhibit cyclical peristaltic contractions induced by maintained distension, must occur independently of the mucosa, or submucosal plexus.

### By what mechanism could 5-HT antagonists cause temporary or sustained inhibition of peristaltic contractions in preparations depleted of endogenous 5-HT?

A major finding of the current study was that in the majority of preparations, the presence of ondansetron and/or SDZ 205-557 caused a rapid blockade of peristaltic contractions. However, in the continuous presence of the drugs, peristaltic contractions gradually recovered had had characteristics not significantly different from controls. The fact that peristalsis contractions still occurred robustly in reserpine-treated animals, with mucosa and submucosal plexus removed confirms our previous work that endogenous 5-HT was not required for the peristalsis. The major new findings of this study is that the antagonists potently inhibited peristaltic contractions in 5-HT depleted preparations showed that the antagonists did not require release of endogenous 5-HT for their inhibitory effects to occur (Figure 7A). One possible explanation for these findings is that 5-HT3 and 5-HT4 receptors may be constitutively active in the absence of any endogenous ligand (i.e., 5-HT). This would explain why the antagonists could still potently inhibit contractions, without any endogenous 5-HT being present in the colon. Indeed, the ligand-gated 5-HT3 receptor (Hu and Peoples, 2008) and the G-protein coupled 5-HT4 (Berthouze et al., 2005) receptor have both been reported to display constitutive activity. If the antagonists reduce this constitutive activity (acting as inverse agonists), this could reduce the background excitability of the enteric neurons that express these receptors and inhibit the neuronal circuitry required for this motor pattern to occur. The reason for the recovery of peristaltic contractions in the continued presence of the antagonists is remarkably similar to the results we reported with hexamethonium, which also temporarily blocked peristalsis, then recovered (Nicholas and Spencer, 2010). We speculate that a sudden reduction of activity in enteric circuits, evoked by antagonists, may be followed by a gradual increase in neuronal excitability that partially compensates, explaining why peristaltic contractions recover. This mechanism would not require endogenous 5-HT.

### Why do the same 5-HT3 and 5-HT4 antagonists have different effects on peristaltic contractions evoked in the same preparation of distal colon?

In a recent study (Sia et al., 2013), we showed that the combined presence of ondansetron and SDZ-205–557 had no significant effect on distension-evoked peristalsis in isolated guinea-pig distal colon. In that study, we evoked peristalsis via a different method to the method using in this current study. In our recent study (Sia et al., 2013), we evoked peristalsis by slow constant fluid infusion, or acutely inserted fecal pellets. In these cases, intraluminal contents were free to be propelled along the colon and expelled by each peristaltic wave. In contrast, in the current study, we recorded peristaltic contractions that were evoked by maintained colonic wall distension, by a fixed fecal pellet that was not free to be propelled along the colon. Interestingly, despite using the same region of colon from the same species, we found that the same antagonists had very different effects on the two different patterns of motor activity evoked. Why the same antagonists, used at the same concentrations, had different effects is unclear. What is clear is that the inhibitory effects we recorded in the current study are unlikely to be due to the blockade of endogenous 5-HT acting on either 5-HT3 or 5-HT4 receptors. This is because the inhibitory effects of these antagonists occurred in colonic preparations that had no detectable 5-HT. This is highly consistent with our recent conclusions and data which showed that endogenous 5-HT was not required for peristalsis (Sia et al., 2013). If the peristaltic contractions were blocked by 5-HT3 and 5-HT4 antagonists because endogenous 5-HT was required for their activation, then depletion of all endogenous 5-HT would be expected to cause the same inhibitory effects. This did not happen. We found no significant deficits in distension-evoked peristaltic contractions in 5-HT depleted preparations. This leads us to believe that the selective 5-HT3 and 5-HT4 antagonists were acting on their cognate receptors to change the excitability of the enteric nervous system, independent of the presence of endogenous 5-HT. Indeed, similar effects were also reported with ondansetron on CMMCs in the isolated mouse colon, where it was found that in mucosa-free colonic preparations treated with reserpine, ondansetron was actually significantly more effective at inhibiting CMMCs (Spencer et al., 2013).

## Conclusions

The findings of the current study show, for the first time, that repetitive peristaltic contractions induced by maintained colonic wall distension can be temporarily inhibited by antagonists of 5-HT3 and 5-HT4 receptors; and these effects occurred equally in preparations that lack any endogenous 5-HT. Only at non-physiologically high concentrations do these antagonists abolish peristaltic contractions. Taken together, none of our findings suggest that the generation of distension-evoked peristaltic contractions in guinea-pig colon requires physiological activation of 5-HT3 or 5-HT4 receptors by endogenous 5-HT. We suggest caution should be exercised when interpreting the initial effects of specific 5-HT3 and 5-HT4 receptor antagonists on the generation of peristaltic reflex motor activity.

## Author contribution

Tiong C. Sia, Sarah J. Nicholas, Malcolm Whiting, Melinda Kyloh, and Nick J. Spencer were involved in performing experiments, study concept and design; acquisition of data; analysis and interpretation of data; drafting of the manuscript. Simon J. Brookes, Phil G. Dinning, John Oliver, David A. Wattchow and were involved in critical revision of the manuscript for intellectual content.

## Grant support

This work was supported by the National Health (NH) and Medical Research Council (MRC) of Australia to Nick J. Spencer (#1025766) and the William Gowan (WG) Norman Fellowship from the Royal Australasian College of Surgeons (RACS) to Tiong Cheng Sia.

### Conflict of interest statement

The authors declare that the research was conducted in the absence of any commercial or financial relationships that could be construed as a potential conflict of interest.
